# When school feels unsafe: bullying victimization, school climate, and adolescent emotional well-being

**DOI:** 10.3389/fpubh.2026.1929270

**Published:** 2026-10-16

**Authors:** Millicent Aziku

**Affiliations:** Faculty of Education, Shaanxi Normal University, Xi'an, China

**Keywords:** bullying victimization, emotional well-being, school belonging, school safety, teacher support

## Abstract

The aim of the current study is to examine the relationships among bullying victimization, school safety, school belonging, teacher support, and emotional well-being using data from the Programme for International Student Assessment (PISA) 2022 with 135,348 adolescents across 15 education systems. The study utilized survey-adjusted hierarchical regression analyses incorporating Balanced Repeated Replication (BRR) weights. Results indicated that bullying victimization was significantly and negatively associated with adolescents’ emotional well-being and perceived school safety. Additionally, school safety and teacher support were positively correlated with school belonging, while school safety, school belonging, and teacher support were positively associated with emotional well-being. Regarding gender differences, the interaction analyses indicated that the association between bullying victimization and school belonging was stronger among female than male adolescents, while the bullying-emotional well-being association did not differ significantly by gender. The findings emphasize the importance of fostering safe, supportive, and inclusive school environments in relation to adolescents’ emotional well-being and experiences of bullying.

## 1 Introduction

To promote the comprehensive development of adolescents, improvement in their mental health and well-being has been consistently discussed among educators and scholars recently (1–4). One critical factor that consistently attracted scholarly attention is bullying, which was consistently identified by prior scholarship as detrimental to adolescents’ well-being and mental health, hence leading to emotional maladjustment (5–7). Adolescents spend a substantial proportion of their productive time at school and with their peers; therefore, experiencing bullying can make the school environment feel unsafe and risky. Adolescents who are exposed to bullying in school have higher probability of experiencing psychological conditions, which are detrimental to their development (8). Such conditions may include anxiety, loneliness, low self-esteem, school disengagement, and suicidal ideation. Consequently, such adolescents may develop a strong aversion to the school environment as an unsafe place since it reminds them of unfavorable interpersonal experiences. School bullying further reduces students’ sense of connectedness within the school environment (9). For adolescents, such emotional experiences are imperative for their development, as they are at a stage characterized by heightened sensitivity to peer relationships, social belonging, and environmental security.

Previous research has studied the influence of bullying victimization on adolescents’ emotional well-being, establishing detrimental associations. However, much of such research had focused on the direct impact of bullying on well-being and other mental health consequences. Few studies have examined how these factors may be interconnected within a theoretically hypothesized psychosocial framework, particularly the relationships involving perceptions of school safety and school belonging (2, 10). Meanwhile, bullying victimization may be associated with adolescents’ perceptions of school safety and sense of school belonging, while these school-climate experiences may also be related to emotional well-being. Also, research has frequently examined school safety, school belonging, and teacher support as isolated correlates rather than as theoretically related dimensions of the school climate. From a public health perspective, understanding how these psychosocial experiences interact is essential because adolescent well-being is shaped not only by individual experiences of victimization but also by broader perceptions of social and environmental support within school settings (25). Examining these factors within an integrated psychosocial framework may, therefore, provide a more comprehensive understanding of the interrelated associations between school environments and adolescents’ emotional well-being.

Furthermore, examining whether associations between bullying victimization and emotional well-being vary by gender is important for understanding potential gender differences in adolescents’ experiences of bullying. This is imperative because existing research demonstrates that there is a gender difference in how adolescents are influenced by peers, indicating that compared to male students, females value relationships more and their emotions are easily shaped by social connectedness compared to males (11). Yet, evidence regarding gender differences in school-climate pathways remains inconsistent, particularly across large international samples. Understanding these differences is important for informing gender-sensitive school-based well-being interventions and public health strategies.

The current study aims to fill these gaps by using data from the Programme for International Student Assessment (PISA) 2022 student questionnaire, including adolescents from different education systems. This study investigates the associations among bullying victimization, perceived school safety, school belonging, teacher support, and emotional well-being within an integrated psychosocial framework. In addition, the study examines whether these associations vary by gender, assessing potential differences between male and female adolescents.

By examining a series of theoretically related associations among bullying victimization, school safety, school belonging, teacher support, and emotional well-being within a psychosocial school-climate framework, this study broadens the literature beyond research focused primarily on the association between bullying victimization and adolescent well-being. Second, it combines several psychological variables of school climate inside a single analytical framework, thereby offering a more holistic understanding of adolescent well-being in educational environments. Finally, by examining gender differences in these associations using a large multinational sample, the study provides evidence on whether adolescents’ relationships with bullying and school-climate experiences vary between male and female students.

## 2 Theoretical background and literature review

Bronfenbrenner (12) bioecological theory suggests that human mental health is a product of person-context interaction and relational processes. This indicates that a person’s development is predicted by the institutions he or she finds him- or herself in, particularly through consistent engagement and participation. In terms of adolescents’ development, this perspective is crucial as this period represents a critical stage where social relationships, school climate, and emotional well-being become deeply interconnected. From a bioecological perspective, adolescents’ development can be described as a process that is driven by interactions with individuals in their ecological systems, such as parents, peers, teachers, etc. Adopting this viewpoint, the current study treats well-being not only as an individual outcome but also as a condition shaped by social and institutional environments. In this study, bullying victimization represents a harmful peer-level experience, school safety reflects the perceived environmental climate, school belonging captures students’ social integration, teacher support represents adult relational support, and emotional well-being reflects adolescents’ psychosocial adjustment.

School bullying has been consistently established by prior scholars as an unfavorable experience, which adversely influences students’ mental health and, hence, emotional well-being. For instance, recent studies revealed a persistent threat to adolescents’ well-being as a result of school bullying, leading to frequent experiences of psychological distress, including anxiety, depressive symptoms, loneliness, and school dropout (9, 10, 13). A more recent finding reinforces the negative link between bullying and adolescents’ well-being by demonstrating that there is a profound inverse association between different forms of bullying, such as verbal, relational, sexual, and physical bullying, and adolescents’ well-being (5). Similarly, another work on peer bullying victimization and psychological distress emphasizes that bullying can undermine adolescents’ emotional adjustment and that school connectedness may be an important protective resource (1).

Meanwhile, the bullying-well-being literature has largely focused on direct associations between victimization and psychological outcomes. Although substantial evidence demonstrates that bullying is associated with poorer mental health, less attention has been devoted to understanding how school-climate factors such as school safety, school belonging, and teacher support are associated with this relationship. This distinction is important because bullying does not occur in isolation from the broader school environment. Adolescents who experience repeated peer victimization may come to perceive school as unsafe, socially threatening, or emotionally unsupportive. Consequently, perceived school safety may constitute an important psychosocial dimension of the school climate that is associated with both bullying victimization and adolescents’ emotional well-being.

One key construct that is pivotal to adolescents’ well-being is environmental safety. For adolescents who are in school, the safety of the school environment is essential for their emotional stability and, hence, well-being, as it assures them that the school environment is both physically and socially secure. From an ecological perspective, unsafe school environments may intensify emotional vulnerability by making everyday school participation stressful (14). Recent school-health work has emphasized that school-related stressors, including bullying and unsafe social environments, can negatively affect adolescent mental health and well-being (15). A review highlights that adolescents’ social and emotional health is an increasing global concern and examines how school climate, including bullying and other social stressors within the school environment, shapes their learning experiences and social and emotional well-being (16). However, school safety is often examined as an independent predictor of adolescent well-being rather than alongside other school-climate factors such as school belonging and teacher support.

Similarly, adolescents’ sense of belonging in school provides a psychosocial pathway in this study. School belonging refers to students’ sense of being accepted, included, respected, and socially connected within the school community. Recent evidence increasingly demonstrates a substantial link between school belonging and adolescent mental health. Allen et al. (17), using a multi-wave prospective cohort study, found that stronger school belonging during adolescence was associated with lower depressive, anxiety, and stress symptoms in young adulthood. A recent review of school belonging similarly highlights the importance of psychological and relational support in strengthening students’ sense of belonging (18). The present study views school belonging not merely as another correlate of emotional well-being but as a key dimension of school climate that is associated with adolescents’ perceptions of school safety and emotional well-being. If bullying makes school feel unsafe, students may become less likely to feel accepted, valued, or connected to their school communities. Consequently, lower levels of school belonging may be associated with poorer emotional well-being among adolescents. This logic is consistent with school connectedness research, which shows that connectedness is a protective factor for adolescent health and well-being (11).

Teacher support is another important dimension of school climate. Positive school climate characteristics such as student-teacher relationships are significantly associated with reduced symptoms of depression and stress and improved self-esteem among adolescents (19). Supportive teachers may provide emotional reassurance, fairness, recognition, and relational security, all of which can strengthen students’ school attachment. Teacher support has a lasting influence on students’ bullying victimization through school belonging, with teacher support negatively predicting bullying victimization among students (20). The current research treats teacher support as an independent school-climate resource that may contribute to emotional well-being and belonging beyond peer experiences. This distinction is important because peer victimization and teacher support represent different social systems within the school ecology. Although supportive teachers may not fully eliminate peer-based victimization, they can still serve as meaningful adult resources that promote belonging and well-being. Teacher support has been shown to buffer the negative effects of bullying victimization on subjective well-being, though the strength of this buffering effect varied across national and social contexts (21). From a practical standpoint, investing in teacher-student relational quality within schools may therefore represent a scalable and cost-effective lever for improving adolescent emotional well-being, particularly among youth who remain vulnerable to peer victimization.

It is important to examine whether associations involving school bullying vary between male and female adolescents because adolescence is characterized by gender differences in emotional distress, peer relationships, and responses to social stress. Recent evidence suggests that the associations among bullying, school climate, and mental health may differ by gender. For example, research on gender differences in bullying and depressive symptoms indicates that different forms of victimization may be associated with different emotional risks for male and female adolescents (22). In contrast, another study found similar associations across genders, suggesting that the nature of relationships within students’ environments may not depend on gender (17). These mixed findings highlight the importance of examining whether the associations among bullying victimization, school safety, school belonging, teacher support, and emotional well-being vary between male and female adolescents.

Despite growing evidence linking bullying victimization to adolescent mental health, several important gaps remain. First, much of the existing literature has focused on the direct association between bullying victimization and psychological outcomes, with comparatively less attention devoted to the broader school-climate context in which bullying occurs. Second, school safety and school belonging are frequently examined as separate correlates of adolescent well-being, despite evidence suggesting that they are closely related dimensions of students’ school experiences. Third, large-scale cross-national research remains limited in examining whether the associations among bullying victimization, school safety, school belonging, teacher support, and emotional well-being differ between male and female adolescents. Addressing these gaps, the present study uses PISA 2022 well-being data to investigate the relationships among bullying victimization, school safety, school belonging, teacher support, and emotional well-being within a psychosocial school-climate framework. The hypothesized framework is presented in Figure 1.

**Figure 1:**
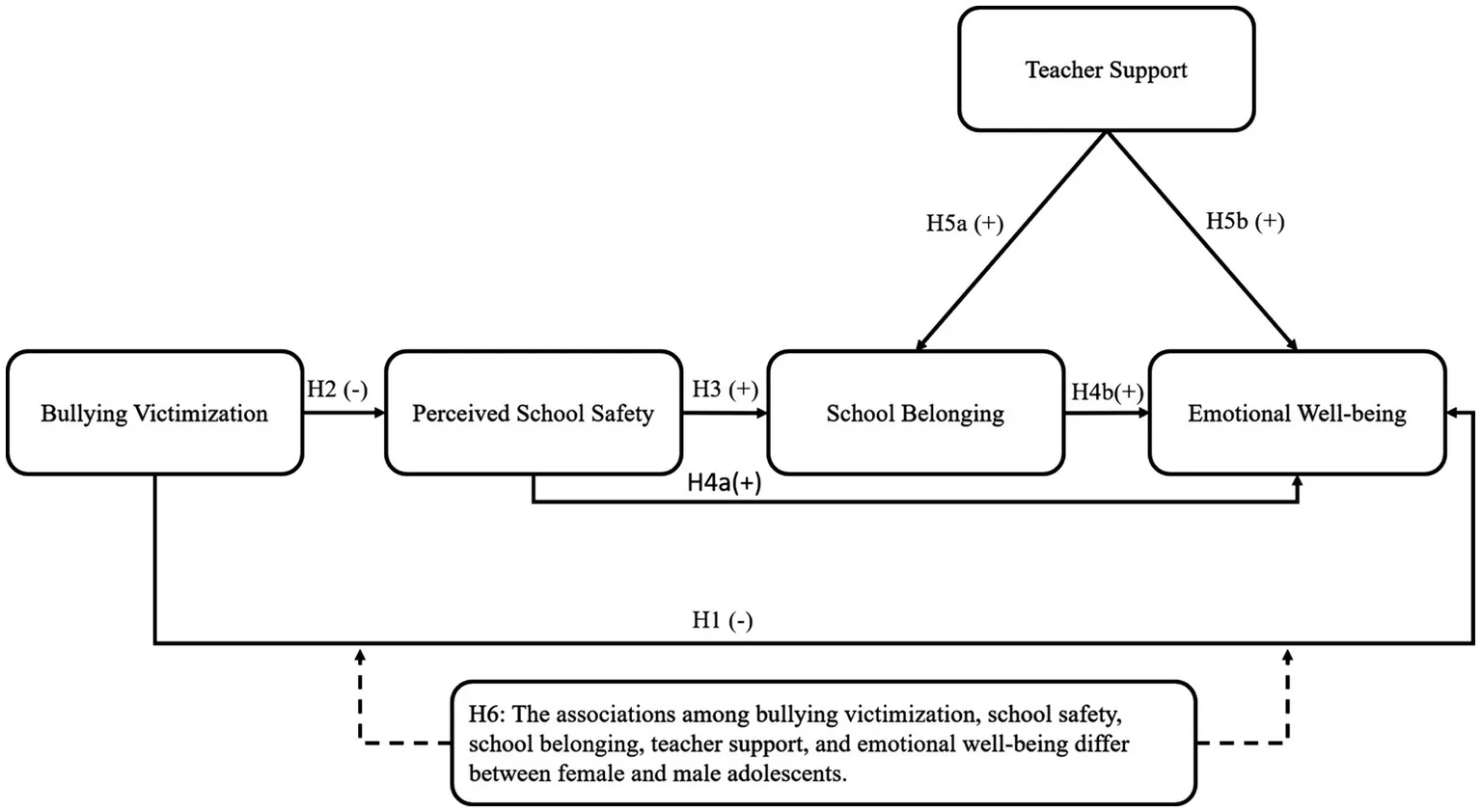
Hypothesized psychosocial pathways. Arrows represent theoretically hypothesized associations examined across a series of separate regression models and do not imply causal or sequential relationships.

*H1*: Bullying victimization is negatively associated with adolescents’ emotional well-being.

*H2*: Bullying victimization is negatively associated with perceived school safety.

*H3*: Perceived school safety is positively associated with school belonging.

*H4a*: Perceived school safety is positively associated with emotional well-being.

*H4b*: School belonging is positively associated with emotional well-being.

*H5a*: Teacher support is positively associated with school belonging.

*H5b*: Teacher support is positively associated with emotional well-being.

*H6*: The strength of the associations among bullying victimization, school safety, school belonging, teacher support, and emotional well-being differs by gender.

## 3 Methods

### 3.1 Data source and participants

To achieve the objectives of the current study, data were obtained from the 2022 wave of the Programme for International Student Avssessment (PISA). PISA is a large-scale international survey conducted by the Organisation for Economic Co-operation and Development (OECD) once every 3 years (23). The data provides comprehensive information on educational experiences of 15-year-olds who are enrolled in schools or educational institutions at the time of the data collection. The PISA questions are used to assess students’ academic and psychosocial experiences across participating education systems using a two-stage stratified sampling design.

In 2022 a well-being questionnaire was administered by 15 education systems, including 135,348 adolescents. The current study leveraged this sample for the statistical analysis since the main outcome of the study is adolescents’ well-being. Out of the total sample, Spain dominated with 22.76%, followed by the United Arab Emirates (UAE) (18.18%), while the rest of the countries each contributed less than 10% of the sample, as shown in Table 1. Participants were predominantly 15- and 16-year-old students enrolled across grades 7 to 12.

**Table 1:** Distribution of students across participating countries (*N* = 135,348).

Country	*n*	%
Brazil	10,798	7.98
Costa Rica	6,113	4.52
France	6,770	5.00
Hong Kong (China)	5,907	4.36
Hungary	6,198	4.58
Ireland	5,569	4.11
Macao (China)	4,384	3.24
Mexico	6,288	4.65
Netherlands	5,046	3.73
New Zealand	4,682	3.46
Panama	4,544	3.36
Saudi Arabia	6,928	5.12
Slovenia	6,721	4.97
Spain	30,800	22.76
United Arab Emirates	24,600	18.18
Total	135,348	100.00

*n*, number of students.

### 3.2 Ethical considerations

This study used secondary, open-access data provided by the OECD through the PISA assessment. All data are fully anonymized, and no identifiable information about individual students or schools is included. As a result, no ethical committee approval or additional informed consent was required for this research.

### 3.3 Measures

The specific PISA 2022 items used to operationalize each construct are presented in Appendix A to facilitate transparency and reproducibility.

#### 3.3.1 Emotional well-being

Emotional well-being was measured using four items assessing the frequency of experiencing symptoms that indicate emotional challenges. Students were asked how often they had certain feelings 6 months prior to the assessment (e.g., Feeling nervous, Difficulties in getting to sleep, feeling anxious, etc.). Responses were recorded on a five-point Likert scale ranging from “Rarely or never” to “About every day.” Items were reverse-coded such that higher scores represented better emotional well-being. Composite scores were calculated using mean item scores. The scale demonstrated acceptable reliability (∝ = 0.84).

#### 3.3.2 Bullying victimization

The current study conceptualizes bullying victimization as students’ experiences of being directly targeted by other students through recurrent peer-based victimization. Accordingly, the measure included six items capturing distinct forms of peer victimization, including deliberate social exclusion, being made fun of, threats, having belongings taken or destroyed, being hit or pushed, and the spreading of nasty rumors. These items were combined into a composite measure to capture general bullying victimization across relational, verbal, threatening, property-related, and physical forms of peer victimization. Responses were recorded on a four-point Likert scale ranging from “Never or almost never” to “Once a week or more.” Higher scores indicated greater bullying victimization. The scale demonstrated acceptable internal consistency (∝ = 0.80).

The study did not include the other items in the PISA bullying-related question block because they capture conceptually distinct experiences. Specifically, involvement in a physical fight does not necessarily indicate victimization, staying home because of feeling unsafe reflects a response to perceived safety rather than a direct experience of peer victimization, and giving money because of threats represents a specific coercive experience. Excluding these items therefore allowed the measure to remain focused on students’ direct experiences of being targeted by peers.

#### 3.3.3 School safety

To measure students’ perception of school safety, they were asked to register their agreement to some statement (e.g., I feel safe in my classrooms at school; I feel safe at other places at school; e.g., hallway, cafeteria, restroom, etc.). Responses were recorded on a four-point Likert scale from “Strongly agree” to “Strongly Disagree.” Items were reverse-coded so that higher scores reflected stronger perceptions of school safety. Reliability was high (∝ = 0.88).

#### 3.3.4 School belonging

School belonging was measured using six items assessing students’ feelings of social acceptance, inclusion, and connectedness at school. Students reported how they feel in school, such as “I feel like I belong at school,” “I feel awkward and out of place in my school,” etc. Responses were recorded on a four-point Likert scale ranging from “Strongly agree” to “Strongly disagree.” After reverse coding where appropriate, higher values indicated stronger school belonging. The scale demonstrated good reliability (∝ = 0.82).

#### 3.3.5 Teacher support

Teacher support was assessed using six items measuring students’ frequency ratings of how often a range of situations occurred during instructional hours (e.g., “The teacher shows an interest in every student’s learning,” “The teacher gives extra help when students need it,” etc.). Responses were reported on a four-point Likert scale ranging from “*Every lesson” to* “Never or almost never.” Higher scores represented greater teacher support. Internal consistency was strong (∝ = 0.84).

#### 3.3.6 Measurement comparability across education systems

The reliability coefficients reported above were calculated on the pooled cross-national sample and therefore indicate that items within each scale correlated consistently across the combined group of respondents, but they do not by themselves establish that the constructs held the same meaning or structure across the 15 participating education systems. Formal measurement invariance testing (e.g., configural, metric, and scalar invariance across countries) was not conducted in the present study, as the analytic strategy relied on standardized observed-variable composites within a survey-adjusted path-analytic framework rather than a multi-group latent-variable model. The PISA instruments used here were developed and field-tested by the OECD through an extensive cross-national adaptation and validation process, including translation/back-translation and pilot testing across participating systems, which provides some prior evidence of cross-national applicability (23). Nonetheless, the absence of study-specific invariance testing means that the pooled associations reported below should be interpreted as reflecting overall patterns across the combined sample rather than assuming strict equivalence of construct meaning at the level of individual education systems.

#### 3.3.7 Control variables

The analyses controlled for age, socioeconomic status (ESCS), grade level, immigrant background, and school absenteeism/skipping behavior. Immigration status distinguished native, second-generation, and first-generation students. School absenteeism reflected whether students had skipped classes during the previous 2 weeks.

### 3.4 Analytical procedure

Before conducting the main statistical analysis, missing values were coded based on OECD procedures. For instance, responses recorded as 7, 8, 9, 96, 97, 98, and 99 were recoded as missing, depending on the item scale. Composite variables were subsequently constructed using mean scores across the relevant items to maximize data retention by allowing respondents with partial item responses to contribute to scale scores when sufficient information was available; no missing-data imputation was performed. Cases with missing values on any variables included in a given regression model were handled through modelwise deletion during estimation. Consequently, the effective analytical sample varied across models according to the availability of the variables required for each model; the number of observations retained for each regression model is reported in Table 2. Following scale construction, all substantive variables were standardized (*z*-scores) to facilitate comparison of effect sizes across models and to allow regression coefficients to be interpreted as standardized effects. All analyses incorporated the PISA final student weights (W_FSTUWT) to produce weighted estimates representative of the student populations within the 15 participating education systems. Because the analyses pooled students across these education systems, the resulting estimates represent overall associations across the combined weighted sample rather than estimates for a single homogeneous international population. Sampling variance was estimated using Balanced Repeated Replication (BRR) with Fay’s adjustment of 0.50 and 80 replicate weights (W_FSTURWT1–W_FSTURWT80) (23).

**Table 2:** Survey-adjusted hierarchical regression models.

Predictor	Model 1 Emotional Well-being (*N* = 125,007)	Model 2 School Safety (*N* = 103,772)	Model 3 School Belonging (*N* = 96,381)	Model 4 Emotional Well-being (*N* = 85,691)
Bullying victimization	−0.257***	−0.193***	−0.204***	−0.190***
School safety	—	—	0.285***	0.111***
School belonging	—	—	—	0.138***
Teacher support	—	—	0.144***	0.054***
Age	−0.227***	−0.100***	−0.010	−0.241***
ESCS	−0.015*	0.101***	0.072***	−0.055***
Grade	0.007	−0.054***	0.042***	0.016
Second-generation immigrant	0.059*	0.171***	−0.014	0.057
First-generation immigrant	0.087**	0.117***	−0.103**	0.112**
School absenteeism	−0.126***	−0.056***	−0.089***	−0.087***
*R^2^*	0.060	0.051	0.154	0.100

Models were estimated using Balanced Repeated Replication (BRR) with Fay’s adjustment (0.50) and PISA student weights. Reference categories were native students (immigration status) and students who did not skip school during the previous 2 weeks. *p < 0.05, **p < 0.01, ***p < 0.001.

The hypothesized relationships were examined using survey-adjusted hierarchical path regression analyses in Stata 19. Rather than estimating a latent-variable structural equation model, the study adopted an observed-variable path-analytic framework based on standardized composite scores. This approach was selected because it accommodates PISA’s complex survey design while allowing the examination of theoretically specified psychosocial pathways.

A sequential hierarchical regression procedure was used to examine theoretically related associations among bullying victimization, school safety, school belonging, teacher support, and emotional well-being. The first model examined the direct association between bullying victimization and emotional well-being while controlling for age, socioeconomic status (ESCS), grade level, immigration background, and school absenteeism. Second, perceived school safety was regressed on bullying victimization and the control variables to examine whether bullying victimization was associated with diminished perceptions of school safety. Third, school belonging was regressed on bullying victimization, school safety, teacher support, and the control variables to examine whether school safety was associated with adolescents’ sense of school connectedness. Finally, emotional well-being was regressed simultaneously on bullying victimization, school safety, school belonging, and the control variables to examine the associations specified in the hypothesized psychosocial framework.

Finally, gender differences in the associations among the study variables were examined by including gender interaction terms in the survey-adjusted regression models. Gender-specific associations were subsequently estimated using Stata’s subpopulation estimation procedures. Multicollinearity diagnostics were calculated using variance inflation factors (VIF) prior to model estimation. The results showed no evidence of problematic multicollinearity. VIF values ranged from 1.02 to 1.27 with an average VIF of 1.09, far below the usually accepted criteria.

## 4 Results

### 4.1 Reliability and preliminary analyses

All study constructs demonstrated acceptable to strong internal consistency (see Table 3). Cronbach’s alpha coefficients were 0.84 for emotional well-being, 0.80 for bullying victimization, 0.88 for school safety, 0.82 for school belonging, and 0.84 for teacher support. Descriptive statistics indicated that bullying victimization was positively skewed as shown in Figure 2, suggesting that most students reported relatively low levels of victimization.

**Table 3:** Reliability statistics for study variables.

Construct	Number of items	Cronbach’s alpha
Emotional wellbeing	4	0.84
Bullying victimization	6	0.80
School safety	4	0.88
School belonging	6	0.82
Teacher support	6	0.84

Multicollinearity diagnostics: VIF = 1.02–1.27; Mean VIF = 1.09.

**Figure 2:**
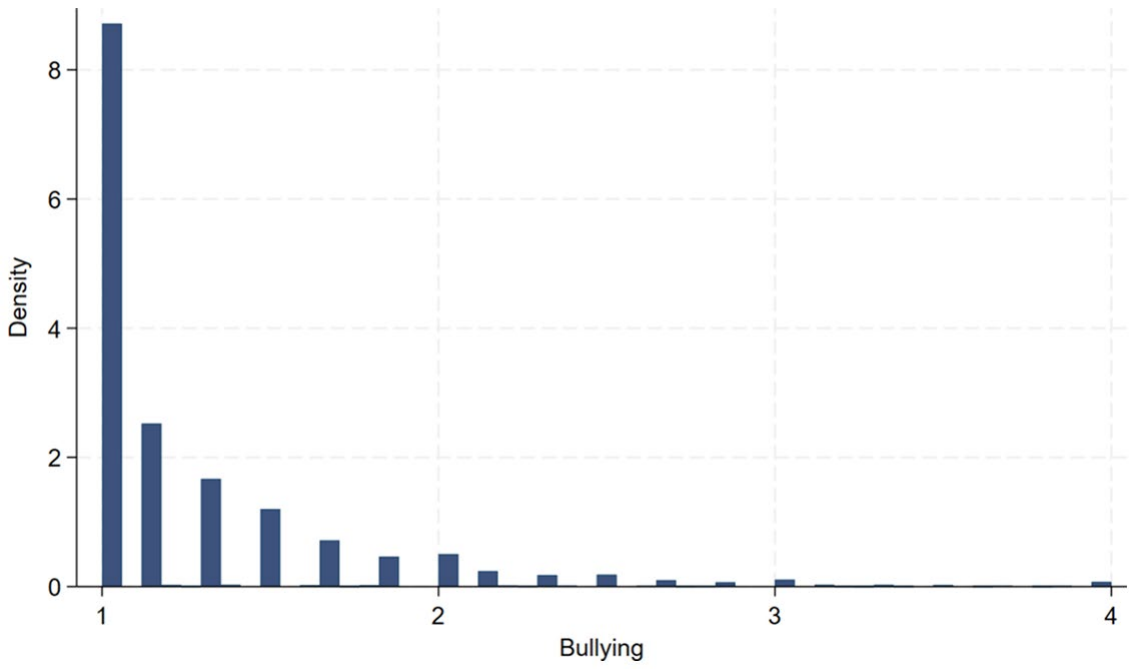
Bullying distribution.

### 4.2 Regression analyses of theoretically related associations

Table 2 summarizes the results, which provide support for H1–H5.

#### 4.2.1 Model 1: Bullying victimization and emotional well-being

For model 1, the $R^{2}$ value was 0.060, indicating that the variables included in the model collectively explained 6% of the variance in emotional well-being. Specifically, the model examined the direct link between bullying victimization and emotional well-being and revealed a significant inverse relationship between the two constructs ( β = −0.257, *p* < 0.001), suggesting that the more students reported being bullied, the weaker the level of emotional well-being reported. Among the control variables, age ( β = −0.227, *p* < 0.001) and school absenteeism ( β = −0.126, *p* < 0.001) were negatively associated with emotional well-being. In contrast, second-generation ( β = 0.059, *p* = 0.015) and first-generation immigrant students ( β = 0.087, *p* = 0.008) reported slightly higher emotional well-being than native students.

#### 4.2.2 Model 2: Bullying victimization and school safety

The second model assessed the relationship between bullying victimization and adolescents’ views of school safety. Bullying victimization was a significant negative predictor of perceived school safety ( β = −0.193, *p* < 0.001). Students who experienced more victimization regarded their school environment to be less safe. Higher socioeconomic position was positively related to safety in school ( β = 0.101, *p* < 0.001). School absenteeism was adversely related to safety in school ( β = −0.056, *p* < 0.001). The model explained 5.1% of the variance in school safety ( $R^{2}$ = 0.051).

#### 4.2.3 Model 3: School safety and school belonging

Model 3 examined the associations of perceived school safety and teacher support with school belonging while controlling for bullying victimization and the covariates. School safety was positively associated with school belonging (*β* = 0.285, *p* < 0.001). Bullying victimization remained negatively linked to school belonging (*β* = −0.204, *p* < 0.001), while teacher support had a significant positive relationship (*β* = 0.144, *p* < 0.001). These results imply that adolescents who perceived their school environments as safe were much more likely to report greater sentiments of school connectivity and belonging. At the same time, bullying victimization independently had a negative correlation with students’ sense of belonging. The model explained 15.4% of the variance in school belonging ( $R^{2}$ = 0.154), representing the strongest explanatory model in the analysis. Collectively, Models 2 and 3 provide support for the proposed psychosocial pathway whereby bullying victimization is associated with diminished perceptions of school safety, which in turn relates to weaker school belonging.

#### 4.2.4 Model 4: Full pathway model predicting emotional well-being

The final model simultaneously included bullying victimization, school safety, school belonging, teacher support, and all control variables to predict emotional well-being. Results revealed that bullying victimization remained negatively associated with emotional well-being ( β = −0.190, *p* < 0.001), even after accounting for school safety, school belonging, and teacher support. Importantly, all three school-climate variables demonstrated significant positive associations with emotional well-being. School belonging emerged as the strongest positive predictor ( β = 0.138, *p* < 0.001), followed by school safety ( β = 0.111, *p* < 0.001) and teacher support ( β = 0.054, *p* < 0.001). These findings indicate that adolescents who perceived their schools as safer, felt a stronger sense of belonging, and reported greater support from teachers experienced higher levels of emotional well-being.

A noteworthy finding was the reduction in the bullying coefficient from β = − 0.257 in Model 1 to β = − 0.190 in Model 4. This attenuation suggests that perceptions of school safety, school belonging, and teacher support collectively account for part of the association between bullying victimization and emotional well-being. Although bullying victimization remained negatively associated with well-being, the findings suggest that positive school-climate experiences are also associated with more favorable emotional well-being. The final model explained 10.0% of the variance in emotional well-being ( $R^{2}$ = 0.100), representing a relatively stronger improvement over the baseline model. Overall, the findings are consistent with the proposed psychosocial framework, with bullying victimization negatively associated with emotional well-being and school safety, school belonging, and teacher support positively associated with emotional well-being and other school-climate experiences.

#### 4.2.5 Gender differences

Formal tests of gender differences indicated that only two associations differed significantly between female and male students. The negative association between bullying victimization and school belonging was stronger among female students than among male students (*β* interaction = −0.049, *p* < 0.001). Similarly, the positive association between perceived school safety and emotional well-being was stronger among female students (*β* interaction = 0.048, *p* = 0.013). However, gender differences were not statistically significant for the associations between bullying victimization and emotional well-being (*p* = 0.324), bullying victimization and school safety (*p* = 0.761), school safety and school belonging (*p* = 0.603), school belonging and emotional well-being (*p* = 0.174), or teacher support and emotional well-being (*p* = 0.589). Thus, H6 received partial support (see Table 4).

**Table 4:** Gender differences in associations (*N* = 75,508).

Association	Interaction *β*	*p*-value	Gender difference?
Bullying → Emotional well-being	−0.019	0.324	No
**School safety → Emotional well-being**	**+0.048**	0.**013**	Yes
School belonging → Emotional well-being	+0.026	0.174	No
Teacher support → Emotional well-being	+0.010	0.589	No
Bullying → School safety	−0.005	0.761	No
**Bullying → School belonging**	**−0.049**	**<0.001**	Yes
School safety → School belonging	−0.008	0.603	No

Models controlled for age, ESCS, grade, immigration background, and school absenteeism. *p < 0.05, **p < 0.01, ***p < 0.001. Bold coefficients indicate statistically significant gender differences.

## 5 Discussion

The present study investigated how bullying victimization is associated with adolescents’ emotional well-being through school-climate experiences, specifically perceived school safety and school belonging, teacher support, while also examining potential gender differences. Although previous studies have consistently established the adverse consequences of bullying on adolescent mental health, less attention has been devoted to understanding how school-climate factors jointly operate within a psychosocial pathway framework. The current study therefore utilized data from the 2022 wave of PISA survey to examine this link. The analysis indicated that bullying victimization was negatively associated with students’ emotional well-being and perceptions of school safety. On the contrary, school safety, school belonging, and teacher support were positively associated with emotional well-being. The findings showed that teacher support related positively to both school belonging and emotional well-being, while several associations appeared stronger among female students than male students.

Supporting the first hypothesis, bullying victimization was inversely correlated with emotional well-being. This finding aligns with a large body of literature indicating that adolescents who experience bullying are more likely to report emotional distress, anxiety, depressive symptoms, loneliness, and lower levels of subjective well-being (5, 9, 13). From a bioecological point of view, bullying is a negative interpersonal experience occurring in the adolescents’ immediate social context. Repeated exposure to victimization may erode perceptions of safety, acceptability, and self-worth, making individuals more emotionally vulnerable. The study further strengthens concerns that bullying should not be treated as a mere disciplinary problem but rather as a serious public-health concern that can undermine adolescents’ psychological functioning and developmental adjustment.

The results suggest that the experience of being bullied was negatively associated to students’ perceptions of safety in their school environment, while school safety had a positive link with belonging. The results of this study support the second and third hypothesis and are consistent with prior research showing that students who were repeatedly victimized tend to develop negative impressions of the larger school environment (15, 16). The issue of school safety is often framed in terms of physical security; however, adolescents’ views of safety also include social and emotional security. Students who are regularly bullied may come to expect future victimization, and hence school environments that are supposed to foster learning and development might be viewed as dangerous. From a bioecological framework, these kinds of experiences may change the ways adolescents engage with their school environment and may decrease the chance of having positive developmental experiences in that setting.

The results further revealed that school safety was positively associated with emotional well-being, supporting hypothesis 4a. This finding aligns with ecological perspectives emphasizing the importance of environmental conditions for healthy adolescent development. When students perceive their schools as safe, they are less likely to experience fear, uncertainty, and social stress, all of which are known contributors to psychological distress. Previous school-health research similarly suggests that perceptions of safety contribute positively to students’ emotional functioning and mental health outcomes (14, 16). The finding highlights that adolescents’ emotional well-being is associated not only with personal characteristics but also with how they perceive the environments in which they learn and interact.

Similarly, the findings support hypothesis 4b by identifying a positive link between school belonging and emotional well-being, which is demonstrated by the strongest positive school-climate factor in the final model. Extant studies have established this pathway, underlining the imperative role of school belonging in shaping adolescents’ mental health (11, 17). Adolescence marks a critical stage in students’ development where they value relations, acceptance, and the attitudes of other people in their immediate environment, if they feel unaccepted and undervalued within their school communities they may develop weak emotions, and psychological insecurity. From a developmental perspective, belonging satisfies fundamental social needs during adolescence, a period characterized by heightened sensitivity to peer relationships and social inclusion. The prominence of school belonging in the current study suggests that fostering stronger feelings of connectedness within schools may represent a particularly effective strategy for promoting adolescent emotional well-being.

Relatedly, teacher support was positively associated with school belonging, supporting hypothesis 5a. This finding is consistent with earlier studies that found that teacher-student interactions are linked to students’ sense of inclusion, engagement, and school belonging (19, 20). Teachers typically are essential non-family individuals in the lives of adolescents and can provide environments marked by fairness, encouragement, and emotional support. Such encounters may enhance students’ feelings of being valued by members of the school community. It is interesting that teacher support remained important after controlling for bullying victimization and school safety, which suggests that positive teacher relationships have a distinctive role in adolescents’ sense of belonging.

The findings additionally supported hypothesis 5b by demonstrating a positive association between teacher support and emotional well-being. Although the magnitude of the relationship was smaller than those observed for school belonging and school safety, the link remained statistically significant after controlling for all other variables in the model. This finding is consistent with previous studies indicating that supportive teachers can promote resilience, emotional security, and positive mental health among adolescents (19, 21, 24). From the perspective of bioecological theory, teacher support represents a positive relational resource within adolescents’ immediate environment. The finding suggests that supportive teacher-student interactions may contribute directly to adolescents’ emotional well-being while also fostering broader positive experiences within the school setting.

Finally, the gender-based analyses indicated that the direction of the associations was generally similar for male and female adolescents, although their magnitude varied for some associations. The association between bullying victimization and emotional well-being did not differ significantly by gender, despite being negative for both groups. In contrast, the positive association between school safety and emotional well-being was significantly stronger among female than male adolescents. The association between bullying victimization and school belonging was also stronger among female adolescents, whereas the gender differences in the associations of school safety and teacher support with school belonging were not statistically significant. These findings provide partial support for the expectation that associations among bullying, school-climate experiences, and emotional well-being may vary by gender. The stronger associations observed for some school-climate dimensions among female adolescents are consistent with prior research suggesting that interpersonal relationships may be particularly salient during female adolescents’ social and emotional development (11, 22). However, the associations were meaningful for both males and females, accentuating the relevance of a supportive school environment for adolescents’ emotional well-being regardless of gender.

### 5.1 Practical implications

The current study has implications for educational stakeholders, school leaders, and public health practitioners seeking to promote adolescent well-being. First, the persistent negative association between bullying victimization and emotional well-being underlines the need for schools to strengthen anti-bullying policies and prevention programs that extend beyond disciplinary approaches to include psychosocial support. Second, the positive roles of school safety and school belonging suggest that fostering emotionally secure and inclusive school environments may be an effective strategy for enhancing adolescent well-being. Schools should therefore invest in initiatives that promote positive peer interactions, inclusiveness, and students’ sense of connectedness to the school community. Furthermore, the significant contributions of teacher support highlight the importance of equipping teachers with the interpersonal and socio-emotional skills necessary to build supportive relationships with students. Finally, the stronger associations observed for some school-climate factors among female students suggest that school-based well-being interventions should consider gender differences in adolescents’ social and emotional experiences. Collectively, these findings indicate that improving adolescent well-being requires a comprehensive school-climate approach that simultaneously addresses bullying, strengthens perceptions of safety, promotes school belonging, and enhances supportive teacher–student relationships.

### 5.2 Limitations and suggestions for future research

The current study has several limitations that need to be considered when making inferences based on the findings. First, its cross-sectional design precludes causal inference and does not establish the temporal ordering of the observed associations. Longitudinal research is needed to examine how these factors relate to one another over time. Second, reliance on PISA 2022 restricted the available variables; important factors such as family support, parenting practices, peer relationships, school anti-bullying policies, and access to mental health services were unavailable. Third, all measures were based on student self-reports and may therefore be affected by recall or social desirability bias. Fourth, bullying victimization was analyzed as a composite measure, preventing examination of subtype-specific associations and whether the observed gender differences partly reflect differences in exposure to particular forms of victimization. Future studies should consider examining bullying subtypes separately to determine whether their associations with school-climate experiences and emotional well-being vary across gender groups.

Fifth, although PISA weights and BRR procedures were applied, between-education-system differences and student nesting within systems were not explicitly modeled. The 15 systems also contributed unequally to the pooled sample, with Spain and the United Arab Emirates accounting for relatively large shares. Moreover, differences in cultural and educational contexts may influence how students perceive bullying, teacher support, school safety, belonging, and emotional well-being. Thus, the findings should be interpreted as pooled patterns across the participating systems rather than relationships that necessarily generalize uniformly across individual systems. Finally, although the PISA instruments were developed and field-tested cross-nationally, formal measurement-invariance testing was not conducted, and reliability was assessed using the pooled sample. Potential differences in construct interpretation across systems therefore cannot be ruled out. Future research should employ longitudinal, multilevel, and measurement-invariance approaches to address these limitations.

## 6 Conclusion

Bullying victimization continues to be a serious threat to adolescents’ mental health, emphasizing the need for more attention to the social and psychological conditions in schools. In a large multinational sample of adolescents, this study found that bullying victimization was connected with lower emotional well-being and decreased perceptions of school safety. In contrast, school safety, school belonging, and teacher support emerged as salient protective factors that were positively related to adolescents’ emotional well-being. Findings also indicated that students with greater perceptions of school safety were more likely to have a better sense of belonging and supportive teacher-student connections were positively associated with school belonging and emotional well-being. Gender subgroup analyses indicated that some associations among bullying victimization, school-climate factors, and emotional well-being were stronger among female than male students, while other associations did not differ significantly by gender. In conclusion, the study highlights the crucial role of creating safe, inclusive, and supportive school environments as a component of holistic strategies for promoting adolescent well-being and addressing the detrimental effects of bullying victimization.

## Data Availability

Data used in this study can be publicly accessed from the PISA website (https://www.oecd.org/en/data/datasets/pisa-2022-database.html), with permission of the OECD.

## References

[1] Ajibewa TA Kershaw KN Carnethon MR Heard-Garris NJ Beach LB Allen NB. Peer bullying victimization, psychological distress, and the protective role of school connectedness among adolescents. BMC Public Health. (2025) 25:2763. doi: 10.1186/s12889-025-24002-6, 40813972 PMC12351988

[2] Arslan G Allen K-A. School victimization, school belongingness, psychological well-being, and emotional problems in adolescents. Child Indic Res. (2021) 14:1501–17. doi: 10.1007/s12187-021-09813-4

[3] Aziku M Huang X Djekourmane D. Multidimensional profiles of adolescent functioning: academic, emotional, and relational patterns. Front Psychol. (2026) 17:1907030. doi: 10.3389/fpsyg.2026.1907030, 42597401 PMC13469240

[4] Liu J Xue M Aziku M. BMI, body image, and subjective wellbeing: a cross-national study of relations among 61,381 15-year-olds. Curr Psychol. (2025) 44:12733–44. doi: 10.1007/s12144-025-07982-x, 30311153

[5] Han Z-Y Ye Z-Y Zhong B-L. School bullying and mental health among adolescents: a narrative review. Translational Pediatrics. (2025) 14:463–72. doi: 10.21037/tp-2024-512, 40225076 PMC11982999

[6] Källmén H Hallgren M. Bullying at school and mental health problems among adolescents: a repeated cross-sectional study. Child Adolesc Psychiatry Ment Health. (2021) 15:74. doi: 10.1186/s13034-021-00425-y, 34906157 PMC8672638

[7] Mullan VMR Golm D Juhl J Sajid S Brandt V. The relationship between peer victimisation, self-esteem, and internalizing symptoms in adolescents: a systematic review and meta-analysis. PLoS One. (2023) 18:e0282224. doi: 10.1371/journal.pone.0282224, 36989220 PMC10058150

[8] Zhao N Yang S Zhang Q Wang J Xie W Tan Y . School bullying results in poor psychological conditions: evidence from a survey of 95,545 subjects. Front Psychol. (2024) 15:1279872. doi: 10.3389/fpsyg.2024.1279872, 38328372 PMC10847557

[9] Donaldson C Morgan K Page N Angel L Moore G. Mental health, bullying and school connectedness: a comparative analysis of school transition at age 11 from within the welsh education system. Br Educ Res J. (2024) 50:1533–55. doi: 10.1002/berj.3985

[10] Hu W Cheng Y Du R. Effects of overt and relational bullying on adolescents’ subjective well-being: the mediating mechanisms of social capital and psychological capital. Int J Environ Res Public Health. (2022) 19:11956. doi: 10.3390/ijerph191911956, 36231258 PMC9565918

[11] Potter JR Yoon KL. Interpersonal factors, peer relationship stressors, and gender differences in adolescent depression. Curr Psychiatry Rep. (2023) 25:759–67. doi: 10.1007/s11920-023-01465-1, 37773480

[12] Bronfenbrenner U. Making human Beings human: Bioecological Perspectives on human Development. Newcastle upon Tyne: SAGE (2005).

[13] Vacca M Cerolini S Zegretti A Zagaria A Lombardo C. Bullying victimization and adolescent depression, anxiety and stress: the mediation of cognitive emotion regulation. Children. (2023) 10:1897. doi: 10.3390/children10121897, 38136099 PMC10742181

[14] Lynch H O’Toole L. "Bronfenbrenner’s bioecological model of human development". In: Routledge Companion to Occupational Therapy. Abingdon: Routledge (2025)

[15] Tsomokos DI Slavich GM. Bullying fosters interpersonal distrust and degrades adolescent mental health as predicted by social safety theory. Nature Mental Health. (2024) 2:328–36. doi: 10.1038/s44220-024-00203-7, 38682098 PMC11052587

[16] Bosacki S Talwar V Lecce S. Critical review: secondary school climate and adolescents’ emotional well-being. Adolescents. (2023) 3:508–23. doi: 10.3390/adolescents3030036

[17] Allen K-A Greenwood CJ Berger E Patlamazoglou L Reupert A Wurf G . Adolescent school belonging and mental health outcomes in young adulthood: findings from a multi-wave prospective cohort study. Sch Ment Health. (2024) 16:149–60. doi: 10.1007/s12310-023-09626-6

[18] Štremfel U Šterman Ivančič K Peras I. Addressing the sense of school belonging among all students? A systematic literature review. Eur J Investig Health Psychol Educ. (2024) 14:2901–17. doi: 10.3390/ejihpe14110190, 39590027 PMC11592540

[19] Podiya JK Navaneetham J Bhola P. Influences of school climate on emotional health and academic achievement of school-going adolescents in India: a systematic review. BMC Public Health. (2025) 25:54. doi: 10.1186/s12889-024-21268-0, 39762815 PMC11706050

[20] Guo J Zhao B Jiang L Pan J. Associations between teacher support, school belonging, and school bullying victimization among Chinese upper elementary school students: a multi-group analysis based on gender. Curr Psychol. (2024) 43:32114–27. doi: 10.1007/s12144-024-06777-w

[21] Oriol X Miranda R Varela J Garcia N. Bullying victimization and subjective well-being in 10- and 12-year-old children from 24 countries: the buffering effect of family and teacher support. Child Indic Res. (2025) 18:1073–100. doi: 10.1007/s12187-025-10226-w

[22] Sun W Gu F Tan B Tang S Tang K Meng J . Gender differences in the association between bullying victimization and depressive symptoms in Chinese children and adolescents. BMC Public Health. (2025) 25:1944. doi: 10.1186/s12889-025-23129-w, 40420087 PMC12105365

[23] OECD. PISA 2022 Technical Report. Paris: OECD Publishing (2023).

[24] Allen K-A Slaten CD Arslan G Roffey S Craig H Vella-Brodrick DA. "School belonging: the importance of student and teacher relationships". In: Kern ML Wehmeyer ML, editors. The Palgrave Handbook of Positive Education. Berlin: Springer International Publishing (2021). p. 525–50.

[25] Wilkins NJ Krause KH Verlenden JV Szucs LE Ussery EN Allen CT . School Connectedness and Risk Behaviors and Experiences Among High School Students—Youth Risk Behavior Survey, United States, 2021. MMWR Supplements, (2023) 72. doi: 10.15585/mmwr.su7201a2, 37104377 PMC10156161

