# Supplementary material for: When school feels unsafe: bullying victimization, school climate, and adolescent emotional well-being
Source: Front Public Health. 2026 Oct 16;14:1929270. doi: 10.3389/fpubh.2026.1929270 (PMC13719485; doi:10.3389/fpubh.2026.1929270)
Supplement: Supplementary file 1 [file Supplementary_file_1.pdf]

Appendix A. PISA 2022 Items, Coding, and Construction of Study Variables

Variable/construct	PISA variable	Item/variable description	Response/coding	Treatment in analysis
Emotional well-being	WB154Q04HA	Feeling depressed	1 = Rarely or never; 2 = About every month; 3 = About every week; 4 = More than once a week; 5 = About every day	Reverse-coded; included
	WB154Q06HA	Feeling nervous	Same as above	Reverse-coded; included
	WB154Q07HA	Difficulties in getting to sleep	Same as above	Reverse-coded; included
	WB154Q09HA	Feeling anxious	Same as above	Reverse-coded; included
Bullying victimization	ST038Q03NA	Other students left me out of things on purpose.	1 = Never or almost never; 2 = A few times a year; 3 = A few times a month; 4 = Once a week or more	Included
	ST038Q04NA	Other students made fun of me.	Same as above	Included
	ST038Q05NA	I was threatened by other students.	Same as above	Included
	ST038Q06NA	Other students took away or destroyed things that belonged to me.	Same as above	Included
	ST038Q07NA	I got hit or pushed around by other students.	Same as above	Included
	ST038Q08NA	Other students spread nasty rumours about me.	Same as above	Included

School safety	ST265Q01JA	I feel safe on my way to school.	1 = Strongly agree; 2 = Agree; 3 = Disagree; 4 = Strongly disagree	Reverse-coded; included
	ST265Q02JA	I feel safe on my way home from school.	Same as above	Reverse-coded; included
	ST265Q03JA	I feel safe in my classrooms at school.	Same as above	Reverse-coded; included
	ST265Q04JA	I feel safe at other places at school (e.g., hallway, cafeteria, restroom).	Same as above	Reverse-coded; included
School belonging	ST034Q01TA	I feel like an outsider (or left out of things) at school.	1 = Strongly agree; 2 = Agree; 3 = Disagree; 4 = Strongly disagree	Included without reversal
	ST034Q02TA	I make friends easily at school.	Same as above	Reverse-coded
	ST034Q03TA	I feel like I belong at school.	Same as above	Reverse-coded
	ST034Q04TA	I feel awkward and out of place in my school.	Same as above	Included without reversal
	ST034Q05TA	Other students seem to like me.	Same as above	Reverse-coded
	ST034Q06TA	I feel lonely at school.	Same as above	Included without reversal
Teacher support	ST267Q01JA	The teachers at my school are respectful towards me.	1 = Strongly disagree; 2 = Disagree; 3 = Agree; 4 = Strongly agree	Included
	ST267Q02JA	If I walked into my classes upset, my teachers would	Same as above	Included

		be concerned about me.		
	ST267Q03JA	If I came back to visit my school three years from now, my teachers would be excited to see me.	Same as above	Included
	ST267Q05JA	When my teachers ask how I am doing, they are really interested in my answer.	Same as above	Included
	ST267Q06JA	The teachers at my school are friendly towards me.	Same as above	Included
	ST267Q07JA	The teachers at my school are interested in students' well-being.	Same as above	Included
Gender	ST004D01T	Student gender	1 = Female; 2 = Male in the source variable; recoded in analysis as 0 = Male, 1 = Female	Binary indicator; used for subgroup and interaction analyses
Age	AGE	Student age	Continuous age variable	Included as a continuous control
Socioeconomic status	ESCS	PISA index of economic, social and cultural status	Continuous standardized PISA index	Included as a continuous control
Grade level	GRADE	Student grade level	Grades 7–12 in the analytical sample	Included as a continuous control
Immigration background	IMMIG	OECD index of immigrant background	Native student; Second-generation	Categorical control; native

			student; First-generation student	students = reference category
School absenteeism/skipping	SKIPPING	Whether the student skipped classes or days of school during the previous two weeks	No classes skipped; At least one class skipped; No response treated as missing	Binary control; no skipping = reference category
